# Raw and Processed Fruit and Vegetable Consumption and 10-Year Coronary Heart Disease Incidence in a Population-Based Cohort Study in the Netherlands

**DOI:** 10.1371/journal.pone.0013609

**Published:** 2010-10-25

**Authors:** Linda M. Oude Griep, Johanna M. Geleijnse, Daan Kromhout, Marga C. Ocké, W. M. Monique Verschuren

**Affiliations:** 1 Division of Human Nutrition, Wageningen University, Wageningen, The Netherlands; 2 National Institute for Public Health and the Environment, Bilthoven, The Netherlands; AgroParisTech, France

## Abstract

**Background:**

Prospective cohort studies have shown that high fruit and vegetable consumption is inversely associated with coronary heart disease (CHD). Whether food processing affects this association is unknown. Therefore, we quantified the association of fruit and vegetable consumption with 10-year CHD incidence in a population-based study in the Netherlands and the effect of processing on these associations.

**Methods:**

Prospective population-based cohort study, including 20,069 men and women aged 20 to 65 years, enrolled between 1993 and 1997 and free of cardiovascular disease at baseline. Diet was assessed using a validated 178-item food frequency questionnaire. Hazard ratios (HR) were calculated for CHD incidence using multivariable Cox proportional hazards models.

**Results:**

During a mean follow-up time of 10.5y, 245 incident cases of CHD were documented, which comprised 211 non-fatal acute myocardial infarctions and 34 fatal CHD events. The risk of CHD incidence was 34% lower for participants with a high intake of total fruit and vegetables (>475 g/d; HR: 0.66; 95% CI: 0.45–0.99) compared to participants with a low total fruit and vegetable consumption (≤241 g/d). Intake of raw fruit and vegetables (>262 g/d vs ≤92 g/d; HR: 0.70; 95% CI: 0.47–1.04) as well as processed fruit and vegetables (>234 g/d vs ≤113 g/d; HR: 0.79; 95% CI: 0.54–1.16) were inversely related with CHD incidence.

**Conclusion:**

Higher consumption of fruit and vegetables, whether consumed raw or processed, may protect against CHD incidence.

## Introduction

Prospective cohort studies have shown that a high consumption of fruit and vegetables is inversely associated with coronary heart disease (CHD) [1], [2]. Fruit and vegetables are rich sources of fiber, vitamins, polyphenols and other bioactive phytochemicals that may contribute to a lower CHD risk [3]. In particular, antioxidants have received considerable attention. Although prospective cohort studies have shown inverse associations between dietary intake of antioxidant vitamins and CHD risk [4], [5], randomized trials using vitamin supplements have failed to demonstrate a beneficial effect [6], [7]. These results may be explained by methodological issues such as the relatively short follow-up period, the high doses of antioxidants compared to habitual diets, and differences in bioavailability of natural and synthetic sources of antioxidants. However, the negative results of these trials could suggest that the protective effect of fruit and vegetables may be due to combined and synergistic effects of the different constituents in their natural food matrix and not to a particular antioxidant [8].

Processing of fruit and vegetables alters their structure and induces significant changes in chemical composition, nutritional value, and bioavailability of bioactive compounds, which may induce different effects on CHD risk. Fruit juices, for example, have a lower content of fiber than raw fruit, but they may be a good source of phytochemicals [9]. Prospective studies that examined intake of citrus fruit juice in relation to risk of CHD [10], and cardiovascular diseases (CVD) [11], respectively, found no association. Vegetables are often cooked before consumption, which induces loss of water-soluble and heat-sensitive bioactive compounds [12], [13]. On the contrary, processing can enhance the availability of bioactive compounds [14]. It has been shown that heat treatment improves the bioavailability of lycopene from tomatoes [15], [16] and carotenoids from carrots [17]. Furthermore, processing could convert folate polyglutamate in vegetables into monoglutamate, which has better bioavailability as well [18], [19].

To the best of our knowledge, no prospective cohort study has focused specifically on raw and processed fruit and vegetable consumption in relation to CHD incidence. Therefore, we investigated the associations of total, raw and processed fruit and vegetable consumption with incident CHD in a population-based follow-up study in the Netherlands.

## Methods

### Population

The present study was conducted in a Dutch population-based cohort: the Monitoring Project on Risk Factors and Chronic Diseases in the Netherlands (MORGEN Study) [20]. Random samples of men and women aged 20 to 59 years were drawn from the municipal registers of two towns in the Netherlands (Amsterdam and Maastricht). In addition, men and women aged 26 to 65 years from the town of Doetinchem, who participated in a similar project between 1987 and 1991 were re-invited to participate in the MORGEN Study. Information on diet, lifestyle, and cardiovascular risk factors was collected between 1993 and 1997. The Medical Ethics Committee of the Netherlands Organization for Applied Scientific Research (TNO) approved the study protocol and all participants signed informed consent form.

Of the total 22,654 participants, we excluded respondents without informed consent for vital status follow-up (*n* = 701), with incomplete dietary assessment (*n* = 72), with reported total energy intake <500 or >4500 kcal per day for women or <800 or >5000 kcal per day for men (*n* = 97), with a history of myocardial infarction or stroke at baseline (*n* = 442) and with self-reported diabetes or using lipid-lowering or anti-hypertensive drugs (*n* = 1,273). This resulted in a study population of 20,069 participants, including 8,988 men and 11,081 women.

### Dietary assessment

Information on habitual food consumption of 178 food items, covering the previous year, was collected using a validated, self-administered, semi-quantitative food frequency questionnaire (FFQ) developed for the Dutch cohorts of the European Prospective Investigation Into Cancer (EPIC) Study [21]. Participants indicated their habitual consumption of food items as absolute frequencies in times per day, per week, per month, per year, or as never, e.g. ‘How often do you habitually eat raw vegetables during a meal in the winter?’. The questions used to assess the frequency of fruit and vegetable consumption were specified as to season and preparation methods. For several food items, additional questions were included about the consumption frequency of different sub-items or about preparation methods using the following categories: always/mostly, often, sometimes, and seldom/never, in answer to questions such as: ‘Which types of vegetables do you eat?’. For 21 food items, mainly vegetables, colored photographs were used to estimate portion sizes. Frequencies per day were multiplied with standard household measures, natural units or indicated portion sizes to obtain grams per day for each food item. The Dutch food composition database of 1996 was used to calculate values for energy and nutrient intakes [22].

The FFQ comprised 35 fruit and vegetable items in total, including 9 raw fruits, 7 raw vegetables, 13 cooked vegetables, 2 vegetable juices/sauces, and 4 fruit juices/sauces. Processed fruits comprised fruit juices and apple sauce that were mainly consumed as industrially produce from concentrates. Processed vegetables comprised home-cooked vegetables including canned and frozen vegetables, and tomato sauce. We did not consider potatoes and legumes, except French beans, as vegetables, because the nutritional value of these food items differs significantly from that of vegetables [22].

The reproducibility of the FFQ after 12 months and relative validity of the FFQ against 12 repeated 24-h recalls for food group and nutrient intake were tested in 63 males and 58 females with mean ages of 42.6 and 49.0 years, respectively [21], [23]. In men, reproducibility of the FFQ after 12 months, expressed as Spearman correlation coefficients, was 0.67 for raw vegetables and 0.69 for cooked vegetables. Similar correlation coefficients for raw and cooked vegetable intake were found in women. The reproducibility for total fruit intake was 0.61 in men and 0.77 in women. The validity against 12 repeated 24-h recalls varied between 0.32 and 0.49 for raw vegetables, 0.21 and 0.41 for cooked vegetables and 0.56 and 0.68 for total fruit intake. Jansen et al validated fruit and vegetable intake by using plasma carotenoids and found that the plasma *β*-cryptoxanthin level was related to the sum of vegetables, fruit and juices (*r* = 0.41 and 0.35 for men and women) and fruit (*r* = 0.32 for both men and women), of which citrus fruit was the major dietary source. Lutein, mainly from green leafy vegetables, was the best indicator of vegetable intake with correlation coefficients of 0.27 and 0.19 for men and women, respectively [24].

### Risk factors

The baseline measurements were previously described in detail by Verschuren *et al* [25]. Body weight, height, and blood pressure of the participants were measured by trained research assistants during a physical examination at a municipal health service site. Non-fasting venous blood samples were collected and total cholesterol concentrations were determined using an enzymatic method. Information on cigarette smoking, educational level, physical activity, use of anti-hypertensive and lipid lowering drugs, past or present use of hormone replacement therapy and the history of myocardial infarction of the participants' parents were obtained through a self-administered questionnaire. Dietary supplement use (yes/no) and alcohol intake were obtained from the FFQ. The most commonly reported dietary supplements included vitamin C (28%), multivitamins (26%) and vitamin B (11%). Alcohol intake was expressed as the number of glasses of beer, wine, port wines, and strong liquor consumed per week. From 1994 onwards, the type, frequency, and duration of physical activity was assessed using a validated questionnaire developed for the EPIC-Study, including questions on leisure time and occupational physical activity [26]. The most frequently reported physical activities were walking, cycling, gardening and sports, of which cycling and sports were activities of at least 4 metabolic equivalents [27]. These four types of physical activities were related to 3-day activity patterns repeated four times [26].

### Ascertainment of fatal and non-fatal events

After enrollment, information on the participants' vital status up to 1 January 2006 was monitored using the municipal population register. For participants who died, information on the primary cause of death was obtained from Statistics Netherlands. The hospital discharge register provided clinically diagnosed AMI admissions by a cardiologist based on the definition of the European Society of Cardiology [28]. It has been shown on the national level that data from the Dutch hospital discharge register can be uniquely matched to a single person for at least 88% of the hospital admissions [29]. In a validation study with an overlap of 33% with our study population, 84% of the AMI cases in the cardiology information system of the University Hospital Maastricht corresponded with AMI cases identified in the hospital discharge register [30]. CHD incidence was defined as the first non-fatal AMI or fatal CHD event, not preceded by any other CHD event. Non-fatal AMI included code 410 of the 9^th^ revision of the International Classification of Diseases [31]. Fatal CHD included ICD-10 codes I20-I25 as the primary cause of death [32]. Where the dates of hospital admission and death coincided the event was considered fatal. Until 1 January 2006, we documented in total 245 incident CHD events, including 211 non-fatal AMI cases and 34 fatal CHD cases.

### Statistical analysis

For each participant, we calculated person time from date of enrollment until the first event (non-fatal AMI or fatal CHD), date of emigration (*n* = 693), date of death or censoring date (1 January 2006), whichever occurred first. Quartiles of intake were computed for each fruit and vegetable group and the lowest quartile was used as the reference category. Hazard ratios (HR) for quartiles of fruit and vegetable consumption were estimated using Cox proportional hazards models. The Cox proportional hazards assumption was fulfilled in all models according to the graphical approach and Schoenfeld residuals. To test *P* for trend of the associations across increasing quartiles of intake, the median values of intake were assigned to each quartile and used as a continuous variable in the Cox model.

Besides an age (continuous) and gender adjusted model, we used a multivariable model that included total energy intake (kcal), smoking status (never, former, current smoker of <10, 10–20, ≥20 cigarettes/d), alcohol intake (never, moderate, and high consumption of >1 glass per day in women and >2 glasses per day in men), educational level (4 categories), dietary supplement use (yes/no), past or present use of hormone replacement therapy (yes/no), family history of AMI before 55y of the father or before 65y of the mother (yes/no) and body mass index (BMI, kg/m^2^). Additionally, we extended the model with dietary covariates including intake of whole grain foods (g/d), processed meat (g/d) and fish (quartiles). Depending on the exposure variable under investigation, we made mutual adjustments for raw or processed fruit and vegetables. Participants with missing data of one or more lifestyle factors were excluded from the analyses of the second and third model (1.2% of the total population). With regard to participants enrolled after 1994, we evaluated whether physical activity was a potential confounder by adding this as a dummy variable to the multivariable model (‘active’ being defined as physically active on at least 5 days a week for 30 minutes or more with an intensity of 4 or more metabolic equivalents). Therefore, we calculated HR with and without adding physical activity into the multivariable model.

Stratified analyses were performed for major cardiovascular risk factors. We examined potential effect modification by age (<50 vs ≥50 y), gender, and smoking status (never vs current) visually as well as by entering cross-product terms into the multivariable model. The log likelihood ratio test was used to compare the models with and without the cross-product terms.

Two-tailed p-values <0.05 were considered statistically significant. Analyses were performed using SAS version 9.1 (SAS Institute).

## Results

Participants with a high intake of fruit and vegetables were more often women, had a higher educational level, were less likely to smoke, used alcohol less often, were more likely to be physically active, used vitamin supplements more often and were more often vegetarian compared to participants with a low intake of fruit and vegetables (**Table 1**). Age and BMI did not differ among quartiles. Participants with a high intake of fruit and vegetables had a higher intake of energy, fruit fiber, vitamin C and potassium compared to participants with a low intake fruit and vegetables (**Table 2**).

**Table 1:** Demographic and lifestyle characteristics by quartiles of fruit and vegetable consumption of 20,069 Dutch participants^1^.

	Quartiles of fruit and vegetable consumption			
	Q1: ≤241 g/d^2^	Q2: 241–346 g/d	Q3: 346–475 g/d	Q4: >475 g/d
Age, y	41.4 (10.7)	41.4 (11.0)	41.6 (11.1)	41.6 (11.5)
Men, %	57.3	45.9	40.2	35.8
Low educational level^3^, %	54.4	47.2	43.7	42.4
Current smokers, %	45.9	37.9	32.6	29.8
High alcohol consumers^4^, %	35.1	31.9	29.6	27.1
Vitamin supplement use, %	25.4	29.7	32	36.4
Physically activity^5^, %	57.8	65.5	70	71.8
Body Mass Index, kg/m^2^	25.1 (4.0)	24.8 (3.8)	24.7 (3.7)	24.7 (3.8)
Serum total cholesterol, mmol/L	5.3 (1.1)	5.3 (1.0)	5.2 (1.0)	5.2 (1.1)
Systolic blood pressure, mmHg	121 (16)	120 (16)	119 (15)	119 (16)
Family history of AMI^6^, %	9.7	8.8	9.2	8.7
Hormone replacement therapy use, %	3.2	4.9	5.5	6.0
Vegetarians, %	1.1	2.7	3.1	5.7
Fish consumers^7^, %	18.4	23.0	27.2	31.2

1 Data are presented as mean (SD) or percentages.

2 100 gram of fruit and vegetables equals 1 medium-sized piece of fruit or 1 cup cut-up raw fruit, fruit juice, cooked vegetables, or 2 cups raw leafy vegetables.

3 Low educational level is defined as primary school and lower, intermediate general education.

4 High alcohol consumption is defined as >1 glass per day in women and >2 glasses per day in men.

5 Physically active on 5 d/wk and ≥0.5 hr/d with an intensity of ≥4 metabolic equivalents. In sub sample of participants enrolled from 1994 onwards (*n* = 15,433).

6 Family history of AMI is defined as occurrence of AMI before 55y of the father or before 65y of the mother.

7 Fish consumption is defined as the highest quartile of fish intake (median: 17 g/d, i.e. ∼1 portion of fish/week).

**Table 2:** Dietary intake by quartiles of fruit and vegetable consumption of 20,069 Dutch participants^1^.

	Quartiles of fruit and vegetable consumption			
	Q1: ≤241 g/d	Q2: 241–346 g/d	Q3: 346–475 g/d	Q4: >475 g/d
Fruit and vegetables, g/d	185 [144–215]	292 [266–319]	404 [374–436]	589 [521–699]
Total energy intake, Kcal	2198 (651)	2243 (653)	2283 (657)	2363 (697)
Total protein, en%	15	15	15	15
Total fat, en%	37	36	35	34
Saturated fatty acids, en%	15	15	15	14
Monounsaturated fatty acids, en%	14	14	14	13
Polyunsaturated fatty acids, en%	7	7	7	7
Trans fatty acids, g/d	4 (2)	4 (2)	4 (2)	4 (2)
Total carbohydrates, en%	43	45	46	48
Mono- disaccharides, g/d	106 (46)	115 (44)	126 (44)	149 (50)
Polysaccharides, g/d	133 (46)	135 (46)	134 (47)	133 (49)
Dietary fiber, g/d	21 (6)	24 (6)	26 (7)	29 (7)
Fruit fiber, g/d	1 (1)	2 (1)	4 (1)	6 (3)
Vegetable fiber, g/d	3 (1)	3 (1)	4 (1)	4 (2)
Dietary fiber from other sources than fruit and vegetables, g/d	18 (6)	18 (6)	19 (6)	18 (6)
Vitamin C, mg/d	62 (16)	88 (15)	113 (19)	164 (42)
Potassium, g/d	3.5 (0.9)	3.7 (0.9)	4.0 (0.9)	4.4 (1.0)

1 Data are presented as mean (SD) or percentages.

The average daily fruit and vegetable intake in our cohort was 378 g/d for the total study population, of which 188 g/d was consumed as raw and 190 g/d as processed fruit and vegetables. The total fruit and vegetable intake was more strongly correlated with raw (*r* = 0.80) than with processed fruit and vegetables (*r* = 0.69). Raw fruit and vegetables were only weakly related to processed fruit and vegetables (*r* = 0.20). The raw fruit and vegetable intake mainly comprised raw fruit, with apples (22% of raw fruit intake) and oranges (18%) as major contributors. Raw vegetables were mainly cucumber (23% of raw vegetable intake) and tomatoes (18%). Processed fruit and vegetables mainly comprised processed fruit, of which citrus juice (24%) and apple juice (22%) were the most important contributors. Processed vegetables were mainly cabbages (24%) and French beans (14%).

The median follow-up time was 10.5 years (interquartile range: 9.2–11.8y). After adjustment for potential confounders, high consumption of total fruit and vegetables was inversely associated with CHD incidence (>475 g/d; HR: 0.66; 95% CI: 0.45–0.99; **Table 3**) compared to participants with a low intake (≤241 g/d). Fruit intake (>328 vs ≤125 g/d; HR: 0.85; 95% CI: 0.58–1.27) as well as vegetable intake (>162 vs ≤96 g/d; HR: 0.88; 95% CI: 0.60–1.30) were inversely related with CHD incidence, although not statistically significant (data not shown). Compared to participants with a low intake, a borderline significant inverse association was observed for a high raw fruit and vegetable intake with CHD incidence (>262 vs ≤92 g/d; HR: 0.70; 95% CI: 0.47–1.04) as well as for a high processed fruit and vegetable intake (>233 vs ≤113 g/d; HR: 0.79; 95% CI: 0.54–1.16).

**Table 3:** Hazard ratios and 95% confidence intervals of CHD incidence by quartiles of fruit. and vegetable intake of 20,069 Dutch participants^1^.

	Quartiles of intake				P for
	Q1^2^	Q2	Q3	Q4	trend
*Total fruit and vegetables*					
Median (g/d)	185	292	404	589	
Cases (n)	88	62	51	44	
Model 1	1.00	0.75 (0.54–1.04)	0.64 (0.46–0.91)	0.58 (0.40–0.84)	0.003
Model 2	1.00	0.85 (0.61–1.19)	0.76 (0.53–1.09)	0.64 (0.43–0.95)	0.02
Model 3	1.00	0.87 (0.62–1.21)	0.79 (0.55–1.13)	0.66 (0.45–0.99)	0.04
*Raw fruits and vegetables* 3					
Median (g/d)	56	127	197	337	
Cases (n)	81	67	54	43	
Model 1	1.00	0.81 (0.59–1.12)	0.67 (0.48–0.95)	0.52 (0.36–0.76)	<.001
Model 2	1.00	0.87 (0.62–1.22)	0.81 (0.56–1.16)	0.66 (0.45–0.98)	0.04
Model 3	1.00	0.89 (0.64–1.25)	0.85 (0.59–1.22)	0.70 (0.47–1.04)	0.08
*Processed fruits and vegetables* 4					
Median (g/d)	87	137	196	301	
Cases (n)	81	70	47	47	
Model 1	1.00	0.99 (0.72–1.36)	0.72 (0.50–1.03)	0.79 (0.55–1.14)	0.10
Model 2	1.00	1.01 (0.73–1.40)	0.77 (0.53–1.11)	0.76 (0.52–1.11)	0.08
Model 3	1.00	1.02 (0.74–1.42)	0.79 (0.55–1.14)	0.79 (0.54–1.16)	0.14

1 Hazard ratios (95% CIs) were obtained from Cox proportional hazards models. Model 1 was adjusted for age and gender (*n* = 20,069). Model 2 was the same as model 1 with additional adjustments for energy intake, alcohol intake, smoking status, educational level, dietary supplement use, use of hormone replacement therapy, family history of MI before 60, BMI (*n* = 19,819). Model 3 was adjusted as model 2 with additional adjustments for intake of fish, whole grain foods and processed meat (*n* = 19,819).

2 Reference group.

3 Additionally adjusted for processed fruit and vegetable intake.

4 Additionally adjusted for raw fruit and vegetable intake.

The associations between total fruit and vegetable intake and CHD did not differ significantly between strata of gender, age, smoking status, educational level, alcohol consumption and dietary supplement use (**table 4**). Correspondingly, we found no evidence for effect modification relative to age, gender or smoking status between raw, processed or total fruit and vegetable intake with CHD incidence. Furthermore, we evaluated whether physical activity was a potential confounder for total fruit and vegetable intake with CHD incidence within participants enrolled from 1994 onwards (*n* = 15,433). HRs for CHD incidence did not change, i.e. 0.51 (95% CI: 0.31–0.84) without physical activity in the model and 0.51 (95% CI: 0.31–0.85) with physical activity included in the model for top vs bottom quartiles of total fruit and vegetable intake.

**Table 4:** Hazard ratios and 95% confidence intervals of CHD incidence by high versus low fruit and vegetable intake among 20,069 participants, stratified by major risk factors^1^.

	Total fruit and vegetable intake		
Risk factor	*n*/cases	Low	High
Gender			
Men	8,988/171	1.00	0.82 (0.59–1.14)
Women	11,061/74	1.00	0.72 (0.44–1.18)
Age			
<50 y	14,655/104	1.00	0.86 (0.56–1.31)
≥50 y	5,414/141	1.00	0.76 (0.53–1.09)
Cigarette smoking			
No	12,701/111	1.00	0.74 (0.50–1.11)
Yes	7,325/133	1.00	0.78 (0.53–1.12)
Educational level			
Low	9,371/148	1.00	0.81 (0.56–1.15)
High	10,061/93	1.00	0.79 (0.51–1.21)
Alcohol consumption			
Never or low	13,865/165	1.00	0.85 (0.61–1.18)
Higher	6,204/80	1.00	0.63 (0.38–1.03)
Dietary supplement use			
No	13,820/180	1.00	0.84 (0.61–1.15)
Yes	6,171/58	1.00	0.63 (0.36–1.11)

1 Hazard ratios (95% CIs) were obtained from Cox proportional hazards models adjusted for age, gender, energy intake, alcohol intake, smoking status, educational level, dietary supplement use, use of hormone replacement therapy, family history of MI before 60, BMI, and for intake of fish, whole grain foods and processed meat.

## Discussion

In the present study, an inverse association of total fruit and vegetable consumption with CHD incidence was observed. This inverse association was present for both raw and processed fruit and vegetables, although these findings did not attain statistical significance.

We had almost complete follow-up for cause-specific mortality as well as for non-fatal events obtained from the hospital discharge register. It has been shown in a validation study that the hospitalized AMI cases corresponded in 84% with AMI cases registered in the hospital discharge register [30]. We may have missed mild myocardial infarction cases that were not hospitalized. However, we expect this to be random and not related to fruit and vegetable intake. Therefore, it is unlikely that this has influenced the relation of fruit and vegetable consumption with CHD incidence.

In the present study, participants with a high intake of fruit and vegetables were more often found to be women who had in general a healthier lifestyle and dietary pattern than men. We observed that confounding was mostly due to age and gender, since additional adjustment for lifestyle and dietary factors did not significantly change the observed associations. However, since fruit and vegetable intake is part of a healthy diet and lifestyle, we cannot rule out residual confounding completely. Furthermore, information was not available on incident diabetes, hypertension or hypercholesterolemia. These participants may have changed their diet intentionally, which may have attenuated the association between fruit and vegetable intake and risk of CHD.

Another limitation of our study was the use of a self-reported FFQ that was assessed at baseline only. Measurement error in self-reported data and changes in dietary habits during follow-up are inevitable and may result in exposure misclassification and attenuation of the results. The weaker associations found for raw and processed fruit and vegetables as well as for subgroup analyses may be a result of the narrower range of intake and limited number of participants. Furthermore, salt is often added to cooked vegetables. Because the FFQ is not a reliable method to assess salt intake, we could not adjust for salt intake and this may have attenuated the results.

We observed an inverse association of total fruit and vegetable intake with CHD incidence, as well as for fruit and vegetables separately. These results are in agreement with meta-analysis data [1], [2]. On the basis of 12 prospective cohort studies, He *et al* observed that participants consuming more than 391 g/d of fruit and vegetables had a 17% lower risk of non-fatal and fatal CHD incidence than those who consumed less than 235 g/d [2]. This corresponds to an 11% lower risk per 100 g/d increase, whereas we observed a 6% lower CHD risk. An earlier meta-analysis of 6 cohort studies showed a weaker association, i.e. a 4% lower risk of CHD incidence for each additional portion fruit or vegetables of 106 gram [1].

In the present study, we found that raw fruit and vegetable consumption was inversely related with CHD incidence. To the best of our knowledge, this is the first prospective cohort study that examined raw fruit and vegetables using an integral approach. However, previous prospective cohort studies have provided partial information on raw fruit and vegetables. With regard to raw vegetables, two prospective cohort studies found inverse associations of salads with fatal CVD [33], [34] and one found no association of higher frequencies of raw vegetable intake with CHD incidence [35]. As concerns raw fruit, prospective cohort studies observed inverse associations of citrus fruit intake and CHD [10], [35], and CVD risk [11]. Raw fruit and vegetables are rich sources of nutrients and bioactive phytochemicals that may have beneficial effects on CHD incidence. It has been demonstrated that a diet rich in fruits and vegetables favourably affects blood pressure levels [36]. These effects may be explained by dietary fiber and potassium, which have been shown to reduce blood pressure levels [37], [38]. Fiber from fruit has been consistently associated with a lower risk of CHD [39], probably through the reduced serum total and LDL cholesterol levels [40], [41]. In a meta-analysis comprising 7 cohort studies, dietary flavonoids lowered CHD mortality by 20% [42]. Flavonoids may also lower CHD risk. In a meta-analysis comprising 7 cohort studies dietary flavonoids lowered CHD mortality by 20% [42].

It is well-known that processing improves the bioavailability and bioaccessibility of bioactive compounds in fruits and vegetables. Heat treatment enhances the bioavailability and strengthens the beneficial health effects of lycopene [15], [16] as well as carotenes [14], [17] and folate [18], [19]. Lycopene, for example, the main carotenoid in tomatoes, accounts for ∼50% of the carotenoids in human blood and is suggested to protect against CVD. Participants with high lycopene concentrations in adipose tissue had a lower myocardial infarction risk [43] and Paran et al reported beneficial effects of tomato extracts on blood pressure in moderate hypertensive participants with uncontrolled hypertension [44].

Processed fruit and vegetables were also inversely related with CHD incidence in the present study. Processed fruits were mainly consumed as fruit juices, which have a lower dietary fiber content but may be good sources of phytochemicals [9]. Moreover, their liquid state affects volume and chewing and may result in decreased satiety and increased energy intake [45]. However, the positive effect of processed fruit was not confirmed in previous prospective cohort studies on citrus fruit juice and CHD [10] and CVD risk [11]. Processed vegetables comprised mainly cooked vegetables. Cooked vegetables also have a generally lower dietary fiber content than raw ones, vitamin C can be lost in cooking water [46] and salt is often added. One study found no association between higher frequencies of baked vegetable intake and CHD incidence [35]. Although fruit juices and cooked vegetables have lost intact cell walls and insoluble fiber [47], these findings suggest that improved bioavailability of bioactive compounds, e.g. flavonoids and carotenoids, may have contributed to the lower risk of CHD incidence.

In conclusion, the results of the present study suggest that a high consumption fruit and vegetables, whether consumed raw or processed, may protect against CHD incidence. Processing of fruit and vegetables affected the observed association with CHD to a small extent.
